# The Clinical Relevance of Methods for Handling Inconclusive Medical Test Results: Quantification of Uncertainty in Medical Decision-Making and Screening

**DOI:** 10.3390/diagnostics8020032

**Published:** 2018-05-09

**Authors:** Johannes A. Landsheer

**Affiliations:** Department of Methodology and Statistics, Faculty of Social and Behavioural Sciences, Utrecht University, 3508 TA Utrecht, The Netherlands; j.a.landsheer@uu.nl; Tel.: +31-30-2534438; Fax: +31-30-2535797

**Keywords:** diagnostic, quantitative tests, reliability, validity, measurement

## Abstract

Background: although the existence of inconclusive medical test results or bio-markers is widely recognized, there are indications that this inherent diagnostic uncertainty is sometimes ignored. This paper discusses three methods for defining and determining inconclusive medical test results, which use different definitions and differ in clinical relevance. Methods: the TG-ROC (two graphs receiver operating characteristics) method is the easiest to use, while the grey zone method and the uncertain interval method require more extensive calculations. Results: this paper discusses the technical details of the methods, as well as advantages and disadvantages for their clinical use. TG-ROC and the grey zone method can help in the acquisition of high rates of diagnostic certainty, but can exclude large groups. The uncertain interval method can prevent decisions that are the most uncertain, invalid and unreliable, while excluding smaller groups. Conclusions: the identification of uncertain test scores is relevant, because these scores indicate the need to obtain better information or to await further developments. The methods presented help to determine inconclusive test scores and can help to reduce erroneous decisions. However, further research and development is desirable.

## Key messages:

- Ignoring the possible uncertainty of test scores is counterproductive as these test scores are the most vulnerable to erroneous decisions.
- The methods presented identify uncertain or inconclusive test scores and can help to prevent decisions for or against the presence of a disease based on these uncertain test scores and, therefore, prevent especially overtreatment.
- Higher rates of diagnostic accuracy are corroborated for the test scores outside the range of most uncertain test scores.
- Further research and development of these methods is desired.

## 1. Introduction

In clinical practice, a diagnostic decision concerns the presence or non-presence of a disease. Uncertainty is the starting point of medical and diagnostic procedures and measurement instruments such as tests and bio-markers are designed to reduce this. However, not all measured results can be classified as either positive or negative. Such inconclusive or uncertain results are an inherent part of the use of diagnostic tests, bio-markers and diagnostic procedures in general. These results have caught the attention of several researchers and are called diagnostically insufficient, inconclusive or grey [1,2], intermediate [3], uncertain [4,5], equivocal, indeterminate or uninterpretable [6] or non-evaluable [7]. Dichotomization of test results, where only decisions for or against the presence of the targeted condition are used, provides no information about these inconclusive test results [8]. Both Simel et al. [6] and Feinstein [8] argued against dichotomization of test scores, as it may cause inconclusive results to be ignored. Both have made early proposals to deal with inconclusive results, using 3 × 2 tables with the uncertain results in addition to decisions for or against the condition to be diagnosed. 

Commonly, measurement uncertainty is defined as the uncertainty concerning the underlying, unobservable true or population value [9] and this uncertainty is generally expressed as standard deviation or a confidence interval. The smaller the interval, the more certain the estimate of the population value, and this certainty is improved by taking larger samples. Its relevance is undebatable for diagnostic measurements, as this uncertainty is an indicator of the generalizability of the estimate based on the measurements. However, in diagnostics the clinician deals with an individual patient or with small groups of similar patients with certain complaints that come to the specific clinical setting. This paper concerns itself with another aspect of uncertainty, namely the inconclusiveness as to whether the patient has the targeted disease or not. This uncertainty can be expressed as the ratio between the estimated probabilities of a test score belonging to the populations of test scores of patients with or without the targeted disease. When this ratio is too close to one, it is impossible to decide whether the patient has the targeted condition or not and the test score is to be considered as uncertain.

This paper concerns three different methods to define this form of uncertainty in a practical way and to express this as a range of uncertain measurement results. The claim of these methods is that it is better not to use these measurement results to make a decision for or against the presence of a targeted condition. This uncertainty is more important when such a decision can lead to serious unwanted consequences and costs which can be avoided by obtaining additional or better information or by awaiting further developments of the disease by watchful waiting or active surveillance. A textbook example of over-diagnosis and overtreatment as a by-product of testing with a relatively weak instrument is the use of prostate specific antigen (PSA) testing when diagnosing prostate cancer [10,11]: the introduction of PSA measurements for diagnosing prostate cancer led to a reduction of the death rate, but also to overdiagnosis and overtreatment. The latter consequences can be avoided by allowing an interval of uncertain test scores instead of applying a single threshold for decisions concerning the presence of the disease.

Schuetz et al. [7] discussed 120 studies that evaluate the coronary computed tomography angiography (CCTA). Non-evaluable results were present in 109 studies. Only 26 studies allowed for the calculation of a 3 × 2 table. In 20 studies, it remained unclear how non-evaluable results were transferred to the client level. Furthermore, when uncertain results were established, the handling of these results varied strongly. The 26 studies that could be evaluated with a 3 × 2 table varied from excluding uncertain results, considering uncertain results positive, or considering uncertain results negative, to applying the intention-to-diagnose principle. The latter approach sees non-evaluable diseased subjects as false positives and non-diseased subjects as false negatives. Applying the intent-to-diagnose principle with the intention not to overestimate sensitivity and specificity tends to underestimate these test accuracy measures [12].

There seems to be little consensus on how to handle inconclusive test results [5,7]. Dichotomization seems the most frequently applied and studied method [13] and a lack of attention to the existence of inconclusive results is frequent practice [7]. However, uncertain results are inherent to diagnostic tests and more so when the test is weaker. This paper discusses three methods that define uncertain test results: the two graphs receiver operating characteristics (TG-ROC) method [3,14,15], the grey zone method [1,2] and the uncertain interval method [4]. All three methods can be applied to quantitative tests, but these three methods differ in their definition of uncertainty and in the way the uncertain scores are determined. They also differ in clinical relevance. Technical details of these methods are included in the Supplementary Materials.

For reasons of clarity, the trichotomization methods discussed here are illustrated by applying these to single diagnostic instruments. In diagnostic practice, the combined use of several diagnostic methods and instruments is usual. An example of applying trichotomization methods to a combination of several diagnostic instruments at the same time is provided for in [4]. Trichotomization methods can also be applied when using diagnostic instruments consecutively, where a test score within the uncertain interval would lead to further testing for the targeted disease.

## 2. Definitions of Diagnostic Uncertainty

**Figure 1:**
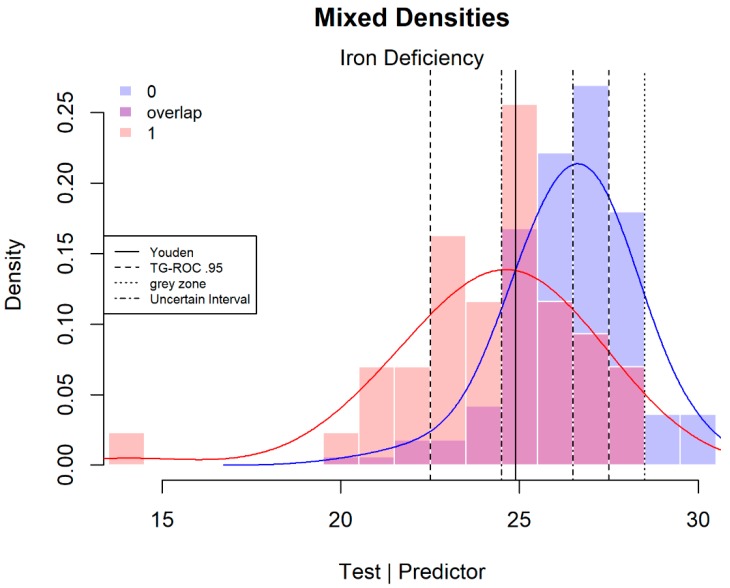
Overlapping test results: mixed densities of reticulocyte haemoglobin content (Chr) in children with (*n* = 43) and without iron deficiency (*n* = 167). The test has a C-statistic (or AUC, area under the curve) of 0.78. Data published in [18]. The lower limits of the two graphs receiver operating characteristics (TG-ROC) and grey zone methods coincide.

In the case of quantitative tests, a smaller or larger part of the overlap between the distributions for subjects with and without the targeted disease can be interpreted as uncertain (Figure 1). Tests that have no overlap and diminish the uncertainty to zero are extremely rare [2]. All three methods discussed here define a larger or smaller interval of the overlapping test scores that are considered diagnostically insufficient and unsuitable for a decision for or against the targeted disease. To the best of my knowledge, these three methods are currently the only ones to define a range of uncertain test scores. There is an alternative three class method, but this method is directed to the specific context of disease states when the identification of more than two classes is relevant and is not concerned with identifying a middle category of uncertain test scores [16,17].

TG-ROC method: the first and most applied method is TG-ROC [3,14,15]. This method defines the most valid outer ranges of test scores that are best used in decisions for or against the disease, indirectly defining a range of test scores that it is better not to use for decision-making. The validity, and therefore the two cut-points, is defined by a chosen high value for sensitivity (*Se*) and specificity (*Sp*), for instance 0.9 or 0.95. Figure 1 shows an application with the lower cut-point determined by an *Se* of 0.95, which defines a lower range where the proportion of false negatives (FN) is limited to 1 − *Se* = 0.05 of test scores of patients with the disease. Likewise, the upper cut-point is defined by an *Sp* of 0.95, limiting the proportion of false positives (FP) to 1 − *Sp* = 0.05 of the test scores of patients without the disease in the upper range. Since *Se* and *Sp* are independent of prevalence of the disease in question, these thresholds are also independent of prevalence. 

When a test has limited strength, the method can exclude a substantial part of the test patients from a decision. Figure 1 shows the mixed densities of patients with and without an iron deficiency. In this example, using a test of intermediate strength (C-statistic or area under the curve (AUC) of 0.78), the method excludes 72.4% of patients. Such a large middle section can still be informative about the disease [2]. The size of the middle section with uncertain scores is larger when the test is weaker [4] or when higher values for *Se* and *Sp* are selected. The use of a 95% or 90% threshold is arbitrary and not inherently dependent on the clinical situation. A more stringent threshold can be chosen when a test is stronger. This example, with a limited number of discrete scores, can only give rough estimates of the desired values for *Se* and *Sp* and the thresholds represent values greater or equal to the desired values for *Se* (1.00 instead of 0.95) and *Sp* (0.952 instead of 0.95).

Grey zone method: a second definition of uncertainty is provided by Coste and Pouchot [2]. They defined a grey zone of inconclusive scores that is linked to the prevalence or pre-test probability of the disease and the clinical requirements for a positive and negative post-test probability. Coste and Pouchot [2] worked out the example with iron deficiency data (Figure 1). Clinical prevalence is used as an estimation of the pre-test probability. This example concerns a sample from a known population with a prevalence estimate of 0.10; this estimate is used by Coste and Pouchot and is also applied here. The reasoning for defining the post-test results is that, due to the severe consequences of failing to detect a patient with iron deficiency, the risk of missing a true patient (negative post-test probability) should be low (0.001), which strongly limits the group of false negatives. Treating a non-patient has few side effects and the positive post-test probability can, therefore, be relaxed to 0.7, allowing for a relatively large group of false positives. 

Coste et al. [1] describe the steps required to define a grey zone. Firstly, the clinical context is analysed and the pre-test probability for the clinical setting is estimated. Secondly, the post-test probabilities required for ruling in or out the diagnostic hypothesis are determined. Thirdly, using the Bayes theorem, the minimum required positive and negative likelihood ratios and/or minimum required *Se* and *Sp* are calculated, and, finally, the grey zone is determined. This final step is essentially the same for both the grey zone and TG-ROC methods, because it makes little difference whether likelihood ratios or *Se* and *Sp* are used, as these statistics are closely related. Technical details for the calculations of the grey zone are described in Supplementary Materials Section 1. 

In the iron deficiency example, this results in a minimum *Se* of 0.99 and a minimum *Sp* of 0.95, corresponding to LR+ ≥ 21 and LR− ≤ 0.009. Figure 1 shows these limits; scores in the range between 23 and 28 are considered diagnostically inconclusive. In this case, the lower limit is equal to the TG-ROC’s lower limit. Nevertheless, the grey zone defined in this way is more restrictive than the arbitrary values chosen for TG-ROC (Figure 1), excluding 185 or 88% of the patients. 

Uncertain interval method: more recently, a third method has been introduced, the uncertain interval method [4]. This method defines the uncertain interval itself, instead of defining the ranges of best applicable test results. The intended clinical relevance of the uncertain interval method is, therefore, the determination of the test scores that it is better not to use for a diagnosis. In Figure 1, the range between 25 and 26 is considered the uncertain interval. The non-parametric approach offers a rough approximation that results in this short range of scores with *Se* = 0.57 and *Sp* = 0.69. There are 39% of deselected scores in the middle section, far less than for the two other methods. Visual inspection of Figure 1 also reveals the highest overlap in this range.

The uncertain interval method defines the test results around the intersection of the two distributions as uncertain, with a specified *Se* and *Sp* for these test results. The intersection is chosen, as this is the point of highest density in the area of overlap where the densities of both distributions are equal. A decision based on a test score near the intersection is no better than a decision based on flipping a coin. The uncertainty is maximal at the point of intersection and decision errors are most concentrated around this point. Schisterman et al. [19] showed that the intersection is equal to the (dichotomous) Youden threshold, which maximizes the sum of *Se* and *Sp*. Consequently, the intersection is also the threshold where the sum of errors (false positives + false negatives) is minimal. (An alternative threshold considered as optimal is the point on the receiver operating curve (ROC) curve closest to the point (0, 1), that is, the upper left corner of the unit square [20,21]. More recently, Perkins and Schisterman showed that the two methods identify the same threshold in certain situations, but when they differ strongly, these researchers found it difficult to consider the ROC (0, 1) threshold as optimal with respect to the overall misclassification rates [22].)

The calculations required for the uncertain interval thresholds are considerably more complicated than those required for the first two methods. The optimization procedure that has been developed under the assumption of a bi-normal distribution is presented in Supplementary Materials Section 2. This approach can easily be extended to other continuous distribution models. A distribution-free implementation of the method is also available.

The uncertain interval is defined as the range of test scores around the intersection that has a low potential to identify patients correctly and is defined using *Se* and *Sp* values for this range. As a default, the value of 0.55 is chosen for both the *Se* and *Sp* of these uncertain test scores. The choice of the default values of the uncertain interval method is discussed in Supplementary Materials Section 3.

The uncertain test scores defined in this way have inherently low validity, but also lead to unreliable decisions. There is a straightforward explanation for the unreliability of decisions based on uncertain test scores: When a measurement is repeated, the second measurement will always deviate slightly from the first and more so when the test is less reliable. These test scores are close to the point of maximum uncertainty and, due to the test’s unreliability, scores lower than this cut-point can become higher than the cut-point when a second measurement is made and vice versa. Decision reliabilities within the uncertain interval are, therefore, low and usually considerably lower than 0.5. A simulation of decision reliabilities that demonstrates this can be found in Supplementary Materials Section 4.

Unsurprisingly, decision reliability is improved in the outer zones. The uncertain interval method does not offer the possibility of specifying values for *Se* and *Sp* for the scores outside the uncertain interval. Nevertheless, it always results in better accuracy for the test scores used in decisions for or against the targeted disease [4]. This is the result of deselecting the test scores around the intersection that offer the highest error rate.

## 3. An Illustrative Example

To demonstrate the capabilities of the three trichotomization methods, we use an empirical dataset to which the bi-normal model can be applied. The dataset has been published in Andrews and Herzberg [23] and is available in the R package *ipred* [24]. The dataset concerns observations of 75 female Duchenne muscular dystrophy (DMD) carriers and 134 female DMD non-carriers. The various methods are demonstrated for the serum creatine kinase (CK), marker for the determination of DMD carriers. The CK marker offers a C-statistic of 0.87. This is not the best marker for this determination, but enables us to compare the three methods. For the CK marker, a Box–Cox transformation (using lambda = −0.34) is applied to obtain a suitable, normal distribution, following the guidelines found in Fluss et al. [25]. The use of the assumption of normal distribution allows for pinning the thresholds to exact points in the distribution and calculating expected values for the different methods. The general advantage of using a parametric approach is that this allows for generalization, even when using samples of limited size, provided that the applied assumption is tenable.

**Figure 2:**
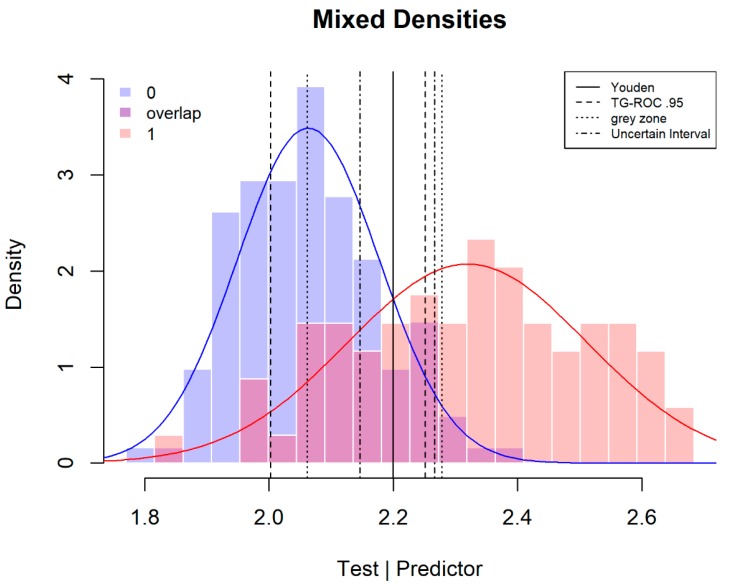
The threshold of the three methods applied to scores of the serum marker CK for the determination of Duchenne muscular dystrophy (DMD) carriers. The receiver operating curve (ROC) (0, 1) threshold is near to the Youden threshold (not displayed).

Figure 2 shows the results for the three methods. For the TG-ROC method, the applied pre-selected value for *Se* and *Sp* is 0.95 (dashed thresholds), for the grey zone the pre-selected values for the positive and negative post-test probabilities are 0.95 and 0.05 (dotted thresholds). For the grey zone method, the sample prevalence (0.36) is used as an uninformed estimation of the pre-test probability. (In general, population estimates of disease prevalence are difficult to obtain for specific groups of patients. When the patient is known to have more or higher relevant risk factors, the higher the prevalence estimate should be taken. In a specific clinical situation, a sample estimate can be a more realistic estimate than the low epidemiological population estimate with no risk factors considered.) For the uncertain interval method the pre-selected values for *Se* and *Sp* of the test scores within the uncertain interval are both 0.6 (dot-dash thresholds).

In Figure 2, the TG-ROC method is the most stringent and cuts out the largest proportion of patients (53%). For the grey zone, the calculation of the desired values of *Se* and *Sp* results in *Se* = 0.91 and *Sp* = 0.97. In this case, the grey zone method cuts out fewer patients than TG-ROC, with a proportion of 42% in the middle zone between the two thresholds. The middle zone of the uncertain interval method is the smallest as it only cuts out 20% of patients. 

Figure 2 also shows the difference in the selection of the two groups: the TG-ROC thresholds mainly deselect patients without the targeted disease (65%) and fewer of the wider distribution of DMD carriers (31%). The thresholds of TG-ROC are dependent on the variances of the two distributions. When the distribution of patients with the targeted condition is wider, fewer of this group of patients are deselected for diagnosis for or against the targeted disease. In the reverse situation, more of these patients would fall in TG-ROC’s middle section. For the grey zone method, the differential selection of patients is dependent on variance differences, the pre-test probability and the difference between the desired values for *Se* and *Sp*. In this case, it deselects more patients without (70%) than with (30%) the targeted disease. The uncertain interval method defines its uncertain interval around the intersection. It is therefore more balanced and deselects 20% of carriers and 21% of non-carriers.

Clinically, what is most interesting are the expected results that the three methods offer for their outer sections (Table 1), assuming the estimated normal distributions are correct.

When trichotomizing, cutting out a considerable part of the overlap between the two distributions means that, for all three methods, the values obtained for *Sp* and *Se* for the two outer regions are larger than when all patients would have been diagnosed. By comparison: when all available data are dichotomized and the sum of *Sp* and *Se* is optimized, the results are 0.73 and 0.88, respectively, with an overall accuracy of 0.83. The results of both the TG-ROC and the grey zone method are somewhat better than those of the uncertain interval method. However, the proportion of diagnosed patients is considerably larger for the uncertain interval method, and the net yield (correctly diagnosed patients as a percentage of all patients) is largest for the uncertain interval method.

There is an issue with both TG-ROC and the grey zone method. None of the pre-selected values for *Sp* and *Se* are obtained for the patients who receive a diagnosis. For TG-ROC, *Sp* and *Se* are 0.86 and 0.93, instead of two times 0.95, while the values for the grey zone method are 0.81 and 0.94 instead of 0.91 and 0.97. The discrepancy between the pre-selected values and the obtained values is described in more detail in Supplementary Materials Section 5. Another issue is that the differential selection of patients results in changed prevalence. For both TG-ROC and the grey zone method, the prevalence has become substantially higher in the selected group of patients in the outer ranges. This creates a logical conundrum for the grey zone method. The post-test probabilities can be recalculated with the values obtained for prevalence, *Se* and *Sp*. For the DMD example, this results in a positive post-test probability of 0.90 and a negative post-test probability of 0.07. These values are still considerable, but also less stringent than the pre-selected values of 0.95 and 0.05 (respectively). Using restrictions that are defined for the full sample does not mean that the same restrictions are obtained for the smaller selected group.

As far as the uncertain interval method is concerned, the values for *Se* and *Sp* are defined for the uncertain interval. As a result, the pre-selected values for *Se* and *Sp* of the uncertain interval are obtained exactly for the bi-normal model. In this example, both *Se* and *Sp* of the uncertain interval are 0.6 for the estimated distributions. When the non-parametric version of the method is used, the pre-selected values are approximated for the given sample.

## 4. The Clinical Relevance of Uncertainty

Knowledge of the uncertainty of an obtained score is relevant because it points to the necessity of obtaining more or better information. In some cases, a more conclusive answer may be obtained from a retest. When available, a better test can be applied; or the obtained results can be combined with the results of additional tests and relevant background information to obtain a more certain decision. Sometimes, it can be sensible to await further developments of the disease. A secondary benefit of the knowledge of inconclusiveness is that it can be communicated to the patient to make him or her more aware of possible developments and promote a possibly necessary second consultation [26]. 

How can the information provided by the three methods help in this respect? The first question is whether it is necessary to obtain high diagnostic certainty. Both the TG-ROC and grey zone methods define high standards for diagnostic certainty, which can exclude large proportions of patients from a decision for or against the targeted disease. Diagnostic certainty can, therefore, be high, but the net yield can be quite low, and more so when a weaker test has been applied. The uncertain interval method focuses on the uncertain results. In most cases, a considerably smaller interval of uncertain test scores is identified. This may provide less diagnostic certainty but a higher net yield. Furthermore, as the method determines an uncertain interval around the intersection, it provides an uncertain interval that contains the most uncertain test scores. The offered diagnostic certainty may, consequently, approximate that offered by the other two methods, while fewer patients are excluded. 

Prevalence also forms a complicating factor. When prevalence differs more strongly from 0.5, it has more influence on post-test probabilities. When random samples are used for the screening of patients, population prevalence is undoubtedly highly relevant. However, patients in a clinical setting are pre-selected through self-selection, referral processes and pre-selection by the practitioner. Consequently, the pre-test expectancy of the clinician can easily be closer to 0.5 than to the (often low) population prevalence. 

The following two tables show the relationship between prevalence and the minimally desired test specifications. Table 2 shows that when *Se* and *Sp* (or LR+ and LR−) are fixed, as is the case in TG-ROC, the positive post-test probability is lower when prevalence is smaller. Conversely, negative post-test probabilities become smaller (i.e., better) when prevalence becomes smaller.

When *Se* equals *Sp* and prevalence is 0.5, the positive post-test probability is equal to *Se* and *Sp*, while the negative post-test probability equals 1 − *Sp* and 1 − *Se*. The TG-ROC and grey zone methods can, therefore, offer the same thresholds.

Table 3 shows the influence of prevalence on the desired levels of *Se* and *Sp* when the post-test probabilities are kept equal. When prevalence is lower, the minimal *Sp* required to achieve the desired post-test probabilities is higher, while the minimal *Se* is lower. Conversely, when prevalence is higher, the demand on *Se* is more significant. When prevalence is 0.5 and the sum of the two post-test probabilities is 1, *Se* and *Sp* are equal to the positive post-test probability.

Only the grey zone method explicitly includes the pre-test expectancy by using pre-test and post-test probabilities. The TG-ROC and uncertain interval methods use prevalence independent indicators *Se* and *Sp*.

Software that is available for the three methods is mentioned in Supplementary Materials Sections 6 and 7.

## 5. Conclusions

The advantages of the knowledge of uncertain test scores: by excluding test results that are prone to erroneous decisions, error rates are reduced for patients with the remaining more conclusive scores. Knowledge that a test result is uncertain indicates the necessity of obtaining more and better information and can prevent exposure of the patient to costs and highly uncertain benefits. Viewed from this perspective, the tendencies that have been reported by Schuetz et al. [7], to ignore unevaluable diagnostic results, as well as the variation in dealing with these scores when they are not ignored, are worrying.

It should be clear that the identification of uncertain measurement scores does not make up for a test or biomarker that has insufficient basic measurement qualities. However, each diagnostic instrument results in a certain number of errors and, thus, in wrong decisions. The latter can be reduced if an identified uncertain test score leads to a decision to use better additional, possibly more expensive, diagnostic tools or to postpone a decision until watchful waiting or the active monitoring of further developments shows otherwise.

Diagnostic certainty and proportion of exclusion: the definitions in the TG-ROC and grey zone methods both aid in the quest for diagnostic certainty. A possible consequence of placing high demands on diagnostic certainty is that a large percentage of the available test scores is deselected for decisions for or against the targeted disease [27], and this becomes more evident when the test is weaker [4]. In contrast to the other two methods, the uncertain interval method focuses on test scores with insufficient validity and reliability and generally excludes fewer patients than both the TG-ROC and grey zone methods. As the uncertain interval method focuses on the part of the overlapping test scores that show the greatest potential for errors, the method may offer a diagnostic certainty that closely approximates that of the other methods, while defining a smaller group with inconclusive results. 

The acceptability of diagnostic insufficiency is not only dependent on the targeted disease, the quality of the test and clinical prevalence, but also on the availability of alternative tests and available treatments with their own success rates and possible, undesirable side effects. The permissible amount of uncertainty can, therefore, vary from setting to setting. It may also be sensible to exclude a large part of the test scores when a test lacks sufficient quality.

Should we use prevalence? Both the TG-ROC and uncertain interval methods use prevalence-independent statistics, while the grey zone definition is connected to a specific clinical situation, using pre- and post-test probabilities. In Bayesian logic, the pre-test probability is not necessarily equal to prevalence. Prevalence is a useful estimate of the pre-test probability, when no further information is available. A complete lack of information is uncommon in diagnostic practice. In most cases, a patient is referred to specifically, and when a patient has explained the symptoms that have led to his or her registration or when information about other tests is available, this additional information influences the expectancy of pre-test probability. It influences expectations, and points to a specific patient spectrum variation or to the applied referral filter [28,29]. Compared to bare prevalence, knowledge of the existence of specific risk factors or the exclusion of other diseases may lead to a considerably higher estimate of the pre-test expectancy of the presence of the targeted condition. Battaglia and Pewsner [27] have argued for the use of prevalence-independent statistics, such as likelihood ratios. Furthermore, when the pre-test probability is close to 0.5, it becomes less relevant whether desired values are chosen for sensitivity and specificity or for positive and negative post-test probabilities. Obtaining prevalence estimates that are appropriate for a given patient can be complex. There is a limited number of studies on the effect of prevalence on bias and variation of accuracy studies and the conclusions are not unambiguous [30]. A more definitive answer is yet to be found.

The three methods all offer the possibility of mapping scores that are lacking in conclusiveness. Without a doubt, using these methods to define a 3 × 2 table is more expedient than ignoring uncertain test scores. This discussion of definitions and methods to determine uncertain test results firstly intends to show the usefulness of knowledge about which test scores have sufficient or insufficient diagnostic certainty. Clearly, there are different ways to define diagnostic certainty and uncertainty, each with their own clinical applicability. Better tests are not easily constructed and these methods for the definition of diagnostic uncertainty help to acquire knowledge about which test scores are best used for decisions for or against a targeted condition. Further research is desirable, and so is discussion about the required amount of diagnostic certainty and how this certainty can be obtained.

## Figures and Tables

**Table 1:** Realized results for the outer sections.

Outer Sections	Proportion	Prevalence	*Sp*	*Se*	Accuracy	Yield
TG-ROC	0.47	0.52	0.86	0.93	0.89	0.42
Grey Zone	0.58	0.42	0.85	0.95	0.89	0.51
Uncertain Interval	0.80	0.36	0.81	0.94	0.85	0.68

**Table 2:** The influence of prevalence on post-test probabilities when *Se* and *Sp* are kept equal.

Pre-Selected Values					Resulting Probabilities	
Prevalence	LR+	LR−	*Se*	*Sp*	Post Positive	Post Negative
0.1	19	0.0526	0.95	0.95	0.6786	0.0058
0.2	19	0.0526	0.95	0.95	0.8261	0.013
0.3	19	0.0526	0.95	0.95	0.8906	0.0221
0.4	19	0.0526	0.95	0.95	0.9268	0.0339
0.5	19	0.0526	0.95	0.95	0.95	0.05
0.6	19	0.0526	0.95	0.95	0.9661	0.0732
0.7	19	0.0526	0.95	0.95	0.9779	0.1094
0.8	19	0.0526	0.95	0.95	0.987	0.1739
0.9	19	0.0526	0.95	0.95	0.9942	0.3214

**Table 3:** The influence of prevalence on the desired levels of *Se* and *Sp* when the post-test probabilities are kept equal.

Pre-Selected Probabilities			Minimum Values Needed			
Prevalence	Post Positive	Post Negative	LR+	LR−	*Se*	*Sp*
0.1	0.95	0.05	171	0.4737	0.5278	0.9969
0.2	0.95	0.05	76	0.2105	0.7917	0.9896
0.3	0.95	0.05	44.3333	0.1228	0.8796	0.9802
0.4	0.95	0.05	28.5	0.0789	0.9236	0.9676
0.5	0.95	0.05	19	0.0526	0.95	0.95
0.6	0.95	0.05	12.6667	0.0351	0.9676	0.9236
0.7	0.95	0.05	8.1429	0.0226	0.9802	0.8796
0.8	0.95	0.05	4.75	0.0132	0.9896	0.7917
0.9	0.95	0.05	2.1111	0.0058	0.9969	0.5278

## Supplementary Materials

The supplementary materials are available online at http://www.mdpi.com/2075-4418/8/2/32/s1.
