# Supplementary material for: The Clinical Relevance of Methods for Handling Inconclusive Medical Test Results: Quantification of Uncertainty in Medical Decision-Making and Screening
Source: Diagnostics (Basel). 2018 May 9;8(2):32. doi: 10.3390/diagnostics8020032 (PMC6023344; doi:10.3390/diagnostics8020032)
Supplement: Supplementary file 1 [file diagnostics-08-00032-s001.pdf]

Supplementary Material

Table of Contents

1. Technical details for the calculation of the Grey zone boundaries 1
2. Optimization procedure for the bi-normal Uncertain Interval method 2
3. Default values of the Uncertain Interval method 7
4. A simulation of decision reliabilities 8
   Design of the simulation study 8
   Decision Reliability 9
5. The discrepancy between the pre-selected values for Se and Sp and the obtained values for TG-ROC and the Grey Zone method 10
6. Available software 13
7. SAS code for the determination of the Grey zone 13
References 15

1. Technical details for the calculation of the Grey zone boundaries

The clinically relevant diagnostic questions which have to be answered beforehand are the prevalence of the disease in the given clinical situation and what post-test probabilities are needed for a given disease to decide for or against starting the treatment of the disease [1]. The idea is that these probabilities are easier clinically defined than likelihood ratios or odds [2].

Combining pre-test probability (p_1) or prevalence with the desired post-test probability (p_2) in the given clinical situation, the likelihood ratio (*LR*) can be calculated, which is defined as the ratio between the post-test odds and the pre-test odds:

$$LR = \frac{\frac{p_2}{(1-p_2)}}{\frac{p_1}{(1-p_1)}} = \frac{(1-p_1)*p_2}{p_1*(1-p_2)}$$

The positive post-test probability for accepting a positive diagnosis and treat the patient without further exploration, and the negative post-test-probability to reject the diagnosis and seek for another explanation of the symptoms, define the necessary levels of *Se* and *Sp*. In the specific clinical setting the prevalence of iron deficiency was set to .1. Consequently, with a positive post-test probability of .7, the desired positive likelihood ratio *LR*+ = ((1 - .1) * .7) / (.1 * (1 - .7)) = 21.   Similarly, with a negative post-test probability of .001, *LR*- = (.9 * .001) / (.1 * .999) = .009.

With the minimum likelihoods known, the minimal required values for are also fixed as *Se* and *Sp* are closely related to *LR*+ and *LR*-:

$$Sp = \frac{1-LR^{+}}{LR^{-}-LR^{+}}$$

$$Se = \frac{LR^{+}(LR^{-}-1)}{LR^{-}-LR^{+}}$$

2. Optimization procedure for the bi-normal Uncertain Interval method

Balancing TP/FN and TN/FP

Figure 1 shows the test scores of non-patients (0; left distribution) and patients (1; right distribution). The two distributions overlap, and as a result, there are both errors and correct decisions for the test scores between an upper (U) and a lower (L) boundaries within this range of overlap:

- True Negatives (TN; patients with a test score that indicates health, while the true status of the patient is healthy);
- False Negatives (FN; healthy patients while their test score indicates the targeted disease);
- True Positives (diseased patients classified as diseased); and

- False Positives (healthy patients classified as diseased).

The upper and lower boundaries are defined in relation to the intersection.

Sensitivity (SE) and specificity (SP) of these test scores within the uncertain interval and around the intersection are defined as usual:

$$SE = \frac{TP}{TP + FN}$$
$$SP = \frac{TN}{TN + FP}$$

Let the distributions of the test scores of non-patients and patients be bi-normal distributed. We try to establish an uncertain interval around the intersection *I* of both curves, so that within this Uncertain Interval both ratios TP/FN and TN/FN are balanced and true decisions and false decisions are about equal. Figure 1 shows this:

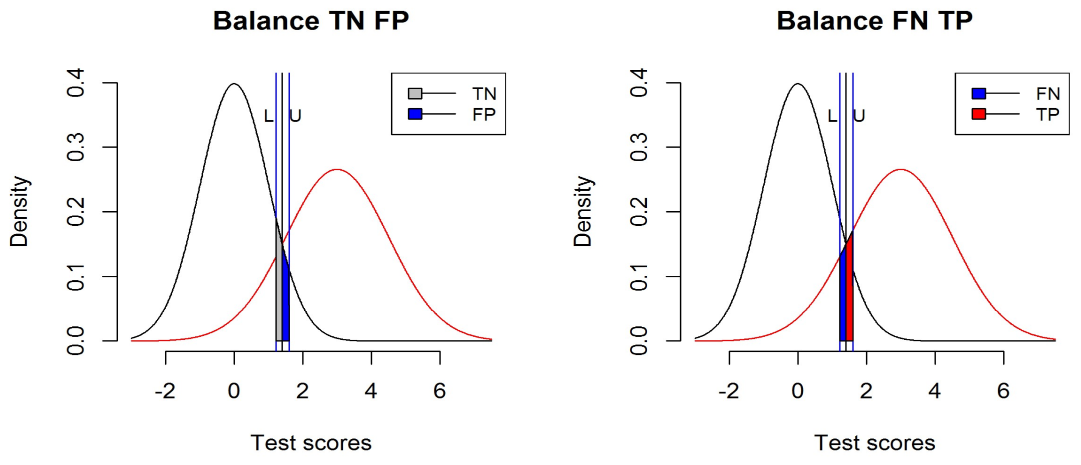

Figure 1: Density plots of normal distributions of Patients without (black) and with the disease (red).

Let φ_0 be the pdf and Φ_0 the cdf of $N(\mu_0, \sigma_0)$, while φ_1 is the pdf and Φ_1 is the cdf of $N(\mu_1, \sigma_1)$, and *I* the point of intersection between the two distributions.

It is assumed that either there is only one intersection, or a second intersection concerns a negligible overlap. If this is not the case, that is, when there are two non-negligible areas of overlap, this means that the test is weak, has interpreational problems and is unfit for application of this method. Schisterman, Perkins, Liu, & Bondell [3] showed that the Youden threshold (and thus the Youden index *J*) occurs at the intersection of both probability density functions, where the overlap is largest. Therefore, the Youden threshold and the uncertain interval are most closely connected. In practice, the estimate of the Youden threshold and the estimate of the main intersection can differ slightly (especially for ordinal test scores), as their calculation is different. In our experience, the intersection based on kernel estimation is slightly more often useful for the estimation of the uncertain interval than the Youden threshold is, but in theory both estimates can be used interchangeably. In most cases they will be the same.

When U is the upper boundary and L is the lower boundary of the uncertain interval, and taking the intersection of both curves as preliminary cut-point, the ratios within the uncertain interval are:

$$\frac{TN}{FP}=\frac{-\Phi_0(L)+\Phi_0(I)}{\Phi_0(U)-\Phi_0(I)}=\frac{Sp}{(1-Sp)}=c_{01}$$
$$\frac{TP}{FN}=\frac{\Phi_1(U)-\Phi_1(I)}{-\Phi_1(L)+\Phi_1(I)}=\frac{Se}{(1-Se)}=c_{11}$$

The constants are provided by choosing a pre-selected value for Se and Sp (default .55). Maximizing the interval (U - L) when using an optimization technique that allows for constraints such as the SLSQP algorithm for nonlinearly constrained gradient-based optimization [4,5], as implemented in R package nloptr [6,7]:

$$min\{-(U-L)^2\}$$

With gradients:

$$\begin{bmatrix}2(U-L) & -2(U-L)\end{bmatrix}$$

Subject to:

$$\frac{-\Phi_0(L)+\Phi_0(I)}{\Phi_0(U)-\Phi_0(I)} \le c_{01}$$

$$\frac{\Phi_1(U)-\Phi_1(I)}{-\Phi_1(L)+\Phi_1(I)} \le c_{11}$$

While the Jacobian matrix of the constraints is:

$$\begin{bmatrix} -\varphi_0(L) & -c_{01}\varphi_0(U) \\ c_{11}\varphi_1(L) & \varphi_1(U) \end{bmatrix}$$

The SLSQP algorithm shows usually good convergence, resulting in a middle section for which both *Se* and *Sp* is very close to the pre-selected value. When this is not the case, the result can be rejected. The optimization fails when U or L are in the extreme tails of the distributions as a result of too demanding values chosen for Se or Sp. Normally, a single minimum exists.

That only a single minimum exists can be shown by optimizing both constraints simultaneously. This leads to an equivalent single optimization function that can be used with general-purpose optimization based on the BFGS algorithm with gradient functions [8].

$$Min\, f(L,U) = \left|-\Phi_0(L) - c_{01}\Phi_1(H) + c_{02}\right| + \left|c_{11}\Phi_1(L) + \Phi_1(H) - c_{12}\right|$$

With constants:

$$c_{01} = Sp/(1-Sp)$$

$$c_{11} = Se/(1-Se)$$

$$c_{02} = (1+c_{01})\Phi_0(I)$$

$$c_{12} = (1+c_{11})\Phi_1(I)$$

And gradient functions:

$$f_L(L,U) = c_{11}\varphi_1(L)\frac{M}{|M|} - \varphi_0(L)\frac{O}{|O|}$$
$$f_U(L,U) = \varphi_1(U)\frac{M}{|M|} - c_{01}\varphi_0(L)\frac{O}{|O|}$$

Where:

$$O = -\Phi_0(L) - c_{01}\Phi_0(U) + c_{02}$$
$$M = c_{11}\Phi_1(L) + \Phi_1(U) - c_{12}$$

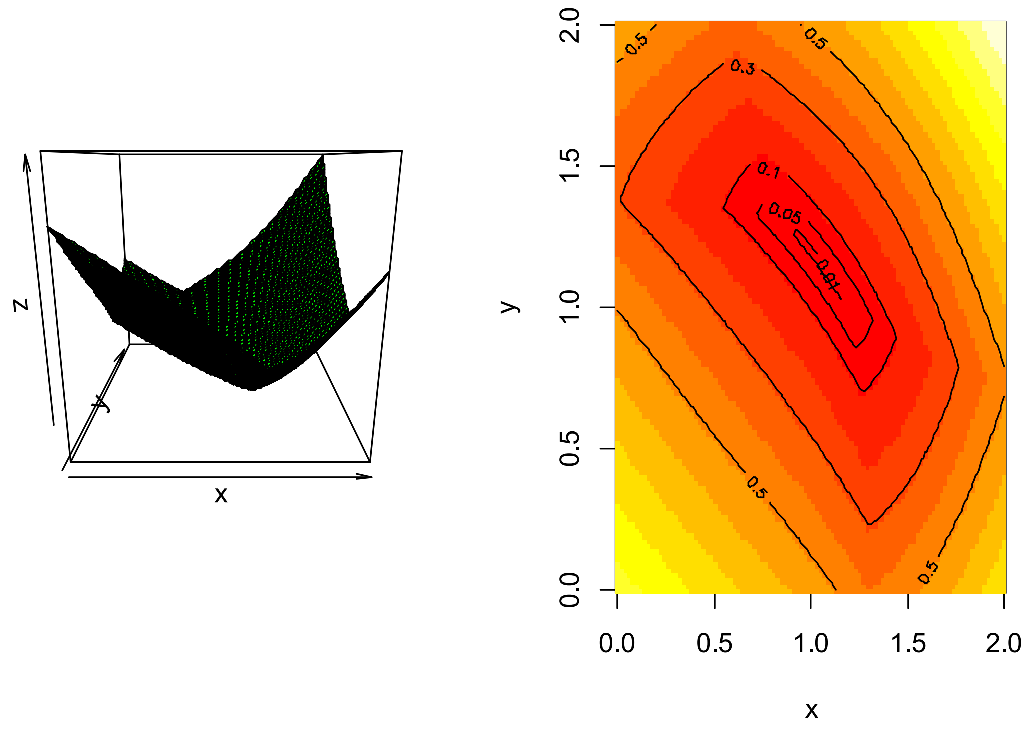

Figure 2: Single minimum for optimization function (example with *N*(0, 1) and *N*(2, .6).

This results in the same solutions as the nlopt optimization provides. Figure 2 shows that there is a single minimum for this optimization. However, optimizing in this way is more vulnerable to start values and therefore the nlopt implementation is preferred.

3. **Default values of the Uncertain Interval method**

The default values for the Uncertain Interval are Se and Sp equal to .55. In that case, the diagnostic odds ratio (DOR) equals 1.49, smaller than a valid small difference between the test scores of subjects with and without the targeted disease [9]. For these test scores, the differences between the pretest probability (prevalence) and posttest probabilities is systematically smaller than 5%, independent of prevalence. When using the default, the test scores within the UI are expected to be statistically insufficient to distinguish 'healthy' from 'diseased' patients in samples of 350 cases or less. The test scores within the uncertain interval are expected to have insufficient validity and reliability and are therefore considered as statistically insufficient for distinguishing patients. A less stringent definition choosing a higher value for *Se* an *Sp* may define test scores that are less uncertain but still diagnostically insufficient. Table A shows results of various choices for *Se* and *Sp*, when normal distributions can be assumed.

Table A. Pre-selected values and obtained values for the Uncertain Interval method. The obtained values are calculated for the selected patients for whom a decision is made, given various test strengths and various pre-selected values for Se and Sp. The reference distribution is *N*(0, 1), while the distribution of the patients with the targeted disease is *N*(*mu*, *sd*).

Patient distribution			Preselected values		Obtained values uncertain interval		Obtained values Outer sections	
**mu**	**sd**	**C**	**Se**	**Sp**	**Se.ui**	**Sp.ui**	**Se.mci**	**Sp.mci**
1	0.5	0.814	0.55	0.55	0.55	0.55	0.733	0.905
1	0.5	0.814	0.55	0.6	0.55	0.6	0.738	0.93
1	0.5	0.814	0.55	0.65	0.55	0.65	0.741	0.953
1	0.5	0.814	0.6	0.55	0.6	0.55	0.74	0.925
1	0.5	0.814	0.6	0.6	0.6	0.6	0.744	0.948
1	0.5	0.814	0.6	0.65	0.6	0.65	0.747	0.967
1	0.5	0.814	0.65	0.55	0.65	0.55	0.747	0.943
1	0.5	0.814	0.65	0.6	0.65	0.6	0.75	0.962
1	0.5	0.814	0.65	0.65	0.65	0.65	0.752	0.979
1	1	0.76	0.55	0.55	0.55	0.55	0.747	0.747
1	1	0.76	0.55	0.6	0.55	0.6	0.771	0.78
1	1	0.76	0.55	0.65	0.55	0.65	0.793	0.821
1	1	0.76	0.6	0.55	0.6	0.55	0.78	0.771
1	1	0.76	0.6	0.6	0.6	0.6	0.806	0.806
1	1	0.76	0.6	0.65	0.6	0.65	0.828	0.848
1	1	0.76	0.65	0.55	0.65	0.55	0.821	0.793
1	1	0.76	0.65	0.6	0.65	0.6	0.848	0.828
1	1	0.76	0.65	0.65	0.65	0.65	0.873	0.873
1	2	0.673	0.55	0.55	0.55	0.55	0.87	0.63
1	2	0.673	0.55	0.6	0.55	0.6	0.903	0.633
1	2	0.673	0.55	0.65	0.55	0.65	0.933	0.633

Patient distribution			Preselected values		Obtained values uncertain interval		Obtained values Outer sections	
**mu**	**sd**	**C**	**Se**	**Sp**	**Se.ui**	**Sp.ui**	**Se.mci**	**Sp.mci**
1	2	0.673	0.6	0.55	0.6	0.55	0.91	0.632
1	2	0.673	0.6	0.6	0.6	0.6	0.939	0.633
1	2	0.673	0.6	0.65	0.6	0.65	0.963	0.632
1	2	0.673	0.65	0.55	0.65	0.55	0.947	0.633
1	2	0.673	0.65	0.6	0.65	0.6	0.969	0.633
1	2	0.673	0.65	0.65	0.65	0.65	0.985	0.632
2	0.5	0.963	0.55	0.55	0.55	0.55	0.899	0.964
2	0.5	0.963	0.55	0.6	0.55	0.6	0.903	0.972
2	0.5	0.963	0.55	0.65	0.55	0.65	0.907	0.979
2	0.5	0.963	0.6	0.55	0.6	0.55	0.905	0.97
2	0.5	0.963	0.6	0.6	0.6	0.6	0.909	0.977
2	0.5	0.963	0.6	0.65	0.6	0.65	0.912	0.983
2	0.5	0.963	0.65	0.55	0.65	0.55	0.911	0.975
2	0.5	0.963	0.65	0.6	0.65	0.6	0.915	0.981
2	0.5	0.963	0.65	0.65	0.65	0.65	0.917	0.987
2	1	0.921	0.55	0.55	0.55	0.55	0.873	0.873
2	1	0.921	0.55	0.6	0.55	0.6	0.885	0.89
2	1	0.921	0.55	0.65	0.55	0.65	0.896	0.907
2	1	0.921	0.6	0.55	0.6	0.55	0.89	0.885
2	1	0.921	0.6	0.6	0.6	0.6	0.901	0.901
2	1	0.921	0.6	0.65	0.6	0.65	0.911	0.918
2	1	0.921	0.65	0.55	0.65	0.55	0.907	0.896
2	1	0.921	0.65	0.6	0.65	0.6	0.918	0.911
2	1	0.921	0.65	0.65	0.65	0.65	0.927	0.927
2	2	0.814	0.55	0.55	0.55	0.55	0.905	0.733
2	2	0.814	0.55	0.6	0.55	0.6	0.925	0.74
2	2	0.814	0.55	0.65	0.55	0.65	0.943	0.747
2	2	0.814	0.6	0.55	0.6	0.55	0.93	0.738
2	2	0.814	0.6	0.6	0.6	0.6	0.948	0.744
2	2	0.814	0.6	0.65	0.6	0.65	0.962	0.75
2	2	0.814	0.65	0.55	0.65	0.55	0.953	0.741
2	2	0.814	0.65	0.6	0.65	0.6	0.967	0.747
2	2	0.814	0.65	0.65	0.65	0.65	0.979	0.752

Se.mci, Sp.mci: Sensitivity and specificity for the test csores in the more certain nterval, the two outer ranges of the Uncertain Interval method
Se.ui, Sp.ui: Sensitivity and specificity for the test csores in the uncertain interval, the middle range of the Uncertain Interval method

In these cases, the obtained values for the uncertain interval (*Se.ui* and *Sp.ui*) are always equal to the preselected values. They may differ when thresholds come to close to the boundaries of the range of overlapping test scores. The obtained values for the outer sections (*Se.mci* and *Sp.mci*) vary, dependent on the pre-selected values and the variance of the patient distribution.

4. A simulation of decision reliabilities

Design of the simulation study

One thousand simulations are executed on an equal number of patients (200) with (*d1*) and without the targeted condition (*d0*), drawn from various bi-normal populations. For *d0* the standard normal distribution is used, while in *d1* both the mean and the standard deviation are varied, representing tests of varying strength as expressed by the population C (concordance or C-statistic) values. The means for the *d1* distribution are 2, 1.2 and .7 and the standard deviations are .7, 1 and 1.6, resulting in concordance values varying from .95 (representing a strong test) to .64 (representing a weak test).

For the study of the reliabilities of the various tests, repeated measurements are simulated by assuming a within-group correlation of .6 between the first and the second measurement. The various cut-points are based on the first measurement and are applied to the second measurement. As the repeated measurements reliability is also dependent on the strength of the test (as indicated by their C-value), the resulting test reliabilities differ considerably (reliabilities ranging from .62 to .83). The reliabilities of the tests are expressed as Pearson's Product Moment Correlations. The decisions reliabilities are evaluated after the various decision methods have been applied. The reliability of the medical decisions is evaluated using Pearson's Phi-coefficient.

Decision Reliability

The repeated measurement reliabilities of both the test and the diagnoses based on Youden, TG-ROC .9 and the bi-normal Uncertain Interval method are shown in table 5. Given a prevalence of .5, the Grey zone method is equal to the TG-ROC approach when the positive post-test probability is .90 and the negative post-test probability is .10.

Table 5. Reliabilities of the diagnoses (means of 1000 simulations)

Populations values			Reliability	Decision Reliabilities			
μ1	σ1	C	Test	Youden	TG-ROC	MCI bi	UI bi
2	0.7	0.95	0.83	0.72	0.74	0.77	0.08
1.2	0.7	0.84	0.73	0.56	0.63	0.64	0.1
0.7	0.7	0.72	0.66	0.47	0.52	0.58	0.12
2	1	0.92	0.8	0.65	0.72	0.72	0.09
1.2	1	0.8	0.71	0.52	0.60	0.63	0.12

Populations values			Reliability	Decision Reliabilities			
μ1	σ1	C	Test	Youden	TG-ROC	MCI bi	UI bi
0.7	1	0.69	0.64	0.45	0.50	0.64	0.18
2	1.6	0.86	0.74	0.59	0.65	0.66	0.1
1.2	1.6	0.74	0.67	0.49	0.53	0.58	0.1
0.7	1.6	0.64	0.62	0.44	0.47	0.54	0.13

TG-ROC: the two outer ranges of the TG-ROC method
MCI bi: More Certain Interval, bi-normal, the two outer ranges of the Uncertain Interval method
UI bi: Uncertain Interval, bi-normal, the middle range of the Uncertain Interval method

The test reliabilities vary from .83 to .62 and are closely related to the test quality as indicated by its C-value. The reliabilities of the diagnoses based on the maximized Youden Index for all test scores are lower and vary between .72 and .44. In comparison to Youden, TG-ROC reliabilities of the decisions based on its two outer ranges are better. The reliabilities of the decisions based on the two outer ranges of the Uncertain Interval method (column MCI bi) are both better compared to those based on Youden and to those based on TG-ROC. They vary from .77 to .54.

As the Youden threshold method and the Uncertain Interval method are closely related, it is possible to use the Youden threshold for calculating the reliabilities of the Uncertain Interval (column UI bi). As expected, the decisions reliabilities based on the test scores in the Uncertain Interval (column UI bi) are very low, with repeated measurements reliabilities varying from .09 to .18.

5. The discrepancy between the pre-selected values for Se and Sp and the obtained values for TG-ROC and the Grey Zone method

For both TG-ROC and the Grey Zone method, the values of *Se* and *Sp* define the small amount of errors (False Negatives and False Positives) that remain in the left and right outer section. However, these are percentages of all patients with (1 – *Se*) and without (1

– *Sp*) the targeted condition. These erroneously diagnosed patients remain in the group of patients who are selected in the outer sections. Because the selected group is smaller than the total group of all patients, the resulting percentages of correctly diagnosed patients (*Sp* and *Se*) in the outer ranges are usually smaller than their pre-selected value. Examples of the expected resulting sensitivity and specificity for the outer ranges of TG-ROC and the Grey Zone method are presented in the. From this table, it can be concluded that it is worthwhile to check the obtained values for the expected distributions, as they may differ considerably from their pre-selected values. The net values for *Se* and *Sp* are not only dependent on their pre-selected values, but also on the strength of the test, the difference of variance in the two distributions, and prevalence.

Table B shows that the obtained values for Se and Sp are usually lower than the pre-selected values (exceptions are highlighted with yellow) and can be extremely low (< .5; highlighted with lila). Obtained LR+ shows most often smaller values (less restrictive), but can be higher because of the changed ratios. Obtained LR- has mostly higher values than the pre-selected values (less restrictive), but can be smaller. When the pre-selected values for Se and Sp differ from each other, the reverse obtained value varies (Sp and Se, respectively). That is because Se determines the lower threshold and Sp the upper threshold.

Table B. Comparison of pre-selected values and obtained values for TG-ROC. The obtained values are calculated for the selected patients for whom a decision is made, given various test strengths and various pre-selected values for Se and Sp. The reference distribution is *N*(0, 1).

Patient distribution			Pre-selected values				Obtained values			
**mu**	**sd**	**C**	**Se**	**Sp**	**LR+**	**LR-**	**Se**	**Sp**	**LR+**	**LR-**
1	0.5	0.814	0.9	0.9	9	0.111	0.741	0.865	5.488	0.299
1	0.5	0.814	0.9	0.95	18	0.105	0.663	0.865	4.912	0.389
1	0.5	0.814	0.9	0.99	90	0.101	0.285	0.865	2.112	0.826
1	0.5	0.814	0.95	0.9	9.5	0.056	0.741	0.919	9.2	0.281
1	0.5	0.814	0.95	0.95	19	0.053	0.663	0.919	8.233	0.366
1	0.5	0.814	0.95	0.99	95	0.051	0.285	0.919	3.541	0.777
1	0.5	0.814	0.99	0.9	9.9	0.011	0.741	0.978	33.006	0.265
1	0.5	0.814	0.99	0.95	19.8	0.011	0.663	0.978	29.537	0.344
1	0.5	0.814	0.99	0.99	99	0.01	0.285	0.978	12.703	0.731
1	1	0.76	0.9	0.9	9	0.111	0.796	0.796	3.891	0.257
1	1	0.76	0.9	0.95	18	0.105	0.838	0.796	4.101	0.203
1	1	0.76	0.9	0.99	90	0.101	0.902	0.796	4.414	0.123
1	1	0.76	0.95	0.9	9.5	0.056	0.796	0.838	4.925	0.244
1	1	0.76	0.95	0.95	19	0.053	0.838	0.838	5.19	0.193
1	1	0.76	0.95	0.99	95	0.051	0.902	0.838	5.585	0.117
1	1	0.76	0.99	0.9	9.9	0.011	0.796	0.902	8.144	0.227
1	1	0.76	0.99	0.95	19.8	0.011	0.838	0.902	8.583	0.179

Patient distribution			Pre-selected values				Obtained values			
**mu**	**sd**	**C**	**Se**	**Sp**	**LR+**	**LR-**	**Se**	**Sp**	**LR+**	**LR-**
1	1	0.76	0.99	0.99	99	0.01	0.902	0.902	9.236	0.108
1	2	0.673	0.9	0.9	9	0.111	0.816	0.371	1.298	0.495
1	2	0.673	0.9	0.95	18	0.105	0.882	0.371	1.402	0.318
1	2	0.673	0.9	0.99	90	0.101	0.962	0.371	1.53	0.102
1	2	0.673	0.95	0.9	9.5	0.056	0.816	0.181	0.996	1.018
1	2	0.673	0.95	0.95	19	0.053	0.882	0.181	1.076	0.654
1	2	0.673	0.95	0.99	95	0.051	0.962	0.181	1.174	0.21
1	2	0.673	0.99	0.9	9.9	0.011	0.816	0.013	0.827	14.351
1	2	0.673	0.99	0.95	19.8	0.011	0.882	0.013	0.893	9.216
1	2	0.673	0.99	0.99	99	0.01	0.962	0.013	0.975	2.962
2	0.5	0.963	0.9	0.9	9	0.111	#			
2	0.5	0.963	0.9	0.95	18	0.105	0.938	0.901	9.505	0.068
2	0.5	0.963	0.9	0.99	90	0.101	0.963	0.901	9.75	0.042
2	0.5	0.963	0.95	0.9	9.5	0.056	0.902	0.946	16.794	0.103
2	0.5	0.963	0.95	0.95	19	0.053	0.938	0.946	17.463	0.065
2	0.5	0.963	0.95	0.99	95	0.051	0.963	0.946	17.913	0.04
2	0.5	0.963	0.99	0.9	9.9	0.011	0.902	0.988	72.973	0.099
2	0.5	0.963	0.99	0.95	19.8	0.011	0.938	0.988	75.881	0.062
2	0.5	0.963	0.99	0.99	99	0.01	0.963	0.988	77.837	0.038
2	1	0.921	0.9	0.9	9	0.111	0.884	0.884	7.638	0.131
2	1	0.921	0.9	0.95	18	0.105	0.927	0.884	8.011	0.082
2	1	0.921	0.9	0.99	90	0.101	0.974	0.884	8.412	0.03
2	1	0.921	0.95	0.9	9.5	0.056	0.884	0.927	12.18	0.125
2	1	0.921	0.95	0.95	19	0.053	0.927	0.927	12.775	0.078
2	1	0.921	0.95	0.99	95	0.051	0.974	0.927	13.415	0.028
2	1	0.921	0.99	0.9	9.9	0.011	0.884	0.974	33.785	0.119
2	1	0.921	0.99	0.95	19.8	0.011	0.927	0.974	35.434	0.075
2	1	0.921	0.99	0.99	99	0.01	0.974	0.974	37.208	0.027
2	2	0.814	0.9	0.9	9	0.111	0.865	0.741	3.344	0.182
2	2	0.814	0.9	0.95	18	0.105	0.919	0.741	3.555	0.109
2	2	0.814	0.9	0.99	90	0.101	0.978	0.741	3.78	0.03
2	2	0.814	0.95	0.9	9.5	0.056	0.865	0.663	2.57	0.204
2	2	0.814	0.95	0.95	19	0.053	0.919	0.663	2.732	0.121
2	2	0.814	0.95	0.99	95	0.051	0.978	0.663	2.905	0.034
2	2	0.814	0.99	0.9	9.9	0.011	0.865	0.285	1.21	0.473
2	2	0.814	0.99	0.95	19.8	0.011	0.919	0.285	1.287	0.282
2	2	0.814	0.99	0.99	99	0.01	0.978	0.285	1.368	0.079

Note #: For distribution $N(2, 0.5)$ with Se = Sp = .9, the lower threshold was higher than the upper threshold and the thresholds are therefore invalid.

Prediction of the obtained values of Se and Sp is complicated, as it is dependent on their pre-selected values, the mean difference, and the difference between the variances. It is also dependent on the prevalence (not shown in this table).

6. Available software

Software for TG-ROC is available in several statistical packages (R, Medcalc and Sigmaplot) [10–12].

Specific software for the determination of the Grey Zone seems to be lacking, but an R function (nomogram) for the first step to calculate sensitivity, specificity, as well as positive and negative likelihood ratios on the basis of desired levels of positive and negative posttest probabilities and prevalence is available in the R package UncertainInterval [13]. When an adequate level for *Se* and *Sp* is defined, commonly available ROC software can be used for the second step. An application based on SAS software [14] for creating different plots for the iron deficiency example is made available in section 7.

An implementation of the Uncertain Interval method is available as the UncertainInterval package for the R language [13]. This package contains two specialized functions for the non-parametric approach, one for the binomial model and a function that can be applied to various continuous distributions.

7. SAS code for the determination of the Grey zone

```
/* With thanks to Joel Coste, who made a basic version of the code available */

/* Generated Code (IMPORT) */
/* Source File: irondef.csv */
/* Source Path: …..
/* Code generated on: 17-10-17 10:47 */

%web_drop_table(WORK.IMPORT);
FILENAME REFFILE '……/irondef.csv';

PROC IMPORT DATAFILE=REFFILE DBMS=CSV OUT=WORK.IMPORT;
	GETNAMES=YES;
RUN;

PROC CONTENTS DATA=WORK.IMPORT;
RUN;

%web_open_table(WORK.IMPORT);
* data totor;
*	set toto;
```

```
DATA IMPORT2;
	SET IMPORT;
	test1=-test; /* use negated values, to make iron deficient patients to have the higher score */
RUN;

proc sort ;
	by test1;
run;

proc freq data=IMPORT2 noprint;
	table test1 /out=bobo;
run;

proc sgplot data=import2;
	* vbar test /group=ref groupdisplay=cluster;
	where ref in (0, 1);

	/* restrict to two groups */
	histogram test1 / group=ref transparency=0.5;

	/* SAS 9.4m2 */
	density test1 / type=kernel group=ref;

	/* overlay density estimates */
run;

proc logistic data=IMPORT2;
	model ref=test1/outroc=bibi ROCEPS=1E-100;
run;

data baba;
	merge bobo(keep=test1 COUNT) bibi (keep=_POS_ _NEG_ _FALPOS_ _FALNEG_ _SENSIT_
		_1MSPEC_);
run;


data baba2;
	set baba;
	preprob=43/210; /* pretest probability = prevalence    */
	preodds=preprob/(1-preprob); * pretest odds
	sp=1-_1MSPEC_;
	se=_SENSIT_;
	lrn=(1-se)/sp;
	lrp=se/(1-sp);
	pospostodds=preodds * lrp;
	pospostprob=pospostodds / (pospostodds+1);
	negpostodds=preodds * lrn;
```

```
        negpostprob = negpostodds / (negpostodds+1);
run;

symbol1 v=dot i=join;

proc gplot data=bibi;
        plot _sensit_*_1mspec_;
        run;
quit;

symbol1 v=dot i=join;

proc sgplot data=baba2;
        series y=lrn x=test1 / lineattrs=(color=blue);
        series y=lrp x=test1 / lineattrs=(color=red);
run;

quit;
symbol1 v=dot i=join;

proc sgplot data=baba2;
        series y=se x=test1 / lineattrs=(color=blue);
        series y=sp x=test1 / lineattrs=(color=red);
run;

quit;
```

References

1. Coste J, Pouchot J. A grey zone for quantitative diagnostic and screening tests. *International journal of epidemiology*. 2003;**32**(2):304–313.

2. Coste J, Jourdain P, Pouchot J. A gray zone assigned to inconclusive results of quantitative diagnostic tests: application to the use of brain natriuretic peptide for diagnosis of heart failure in acute dyspneic patients. *Clinical chemistry*. 2006;**52**(12):2229–2235.

3. Schisterman EF, Perkins NJ, Liu A, Bondell H. Optimal cut-point and its corresponding Youden Index to discriminate individuals using pooled blood samples. *Epidemiology*. 2005;73–81.

4. Kraft D. A software package for sequential quadratic programming. *Forschungsbericht- Deutsche Forschungs- und Versuchsanstalt fur Luft- und Raumfahrt*. 1988;

5. Kraft D. Algorithm 733: TOMP–Fortran modules for optimal control calculations. *ACM Transactions on Mathematical Software (TOMS)*. 1994;**20**(3):262–281.

6. Powell MJ. A view of algorithms for optimization without derivatives. *Mathematics Today-Bulletin of the Institute of Mathematics and its Applications*. 2007;**43**(5):170–174.

7. Johnson SG. NLopt - AbInitio [Internet]. 2014 [cited 2016 Dec 8]. Available from: http://ab-initio.mit.edu/wiki/index.php/NLopt

8. Nash JC. Compact numerical methods for computers: linear algebra and function minimisation. CRC Press; 1990.

9. Chen H, Cohen P, Chen S. How big is a big odds ratio? Interpreting the magnitudes of odds ratios in epidemiological studies. *Communications in Statistics—Simulation and Computation®*. 2010;**39**(4):860–864.

10. Brasil P. DiagnosisMed: Diagnostic test accuracy evaluation for medical professionals [Internet]. 2010. Available from: https://r-forge.r-project.org/R/?group_id=258

11. Schoonjans F. ROC curve analysis with MedCalc [Internet]. *MedCalc* 2018 [cited 2018 Jan 16]. Available from: https://www.medcalc.org/manual/roc-curves.php

12. Sigmaplot. SigmaPlot: ROC Curves Analysis [Internet]. 2018 [cited 2018 Jan 16]. Available from: http://www.sigmaplot.co.uk/splot/products/sigmaplot/productuses/prod-uses42.php

13. Landsheer J.A. CRAN - Package UncertainInterval [Internet]. 2017 [cited 2017 Feb 14]. Available from: https://cran.r-project.org/web/packages/UncertainInterval/index.html

14. Sas Institute Inc. SAS 9.4 Help and Documentation. Cary, NC: SAS institute Inc; 2017.
